# The Problem of Investigating Causal Relationships Between Cognitive and Evaluative Variables

**DOI:** 10.2196/45570

**Published:** 2023-11-22

**Authors:** Uwe Konerding

**Affiliations:** 1 Trimberg Research Academy University of Bamberg Bamberg Germany; 2 Department of Psychology and Psychotherapy Faculty of Health University of Witten/Herdecke Witten Germany

**Keywords:** social influence, physician rating websites, patient satisfaction, eHealth literacy

In a manuscript published in the *Journal of Medical Internet Research*, Guetz and Bidmon [1] present two empirical studies to test a model predicting the intention to use physician rating websites. According to this model, social influence affects intention, and this effect is mediated by credibility and performance expectation, whereby credibility influences performance expectation. In study 1, the authors manipulated social influence experimentally and assessed the remaining three variables using questionnaires. In study 2, they assessed all four variables using questionnaires following a cross-sectional design. They analyzed the data from both studies by computing bivariate correlations as well as linear regressions from credibility on social influence; from performance expectation on social influence and credibility; and from intention on social influence, credibility, and performance expectations.

In study 1, the authors found no significant bivariate correlation between social influence and intention. The overall tests for the three regressions were significant, but the paths from social influence to performance expectations and to intentions as well as the path from credibility to intentions were not significant. The authors interpreted these results as evidence for a nondirect but mediated effect from social influence on intention. In study 2, all tests yielded a significant result, with the bivariate correlation between social influence and intention being larger than the corresponding regression coefficient in the last linear regression. When trying to interpret these results, the authors stated that the cross-sectional approach is “less appropriate to indicate the direction of proposed effects” and tried to avoid terms with a causal meaning when describing their results. However, in the Discussion section, they interpreted these results as evidence for their model.

The authors’ interpretations merit some comments. By definition, an effect between two variables can only be mediated by other variables when this effect exists without the mediating variables being considered. Therefore, the results of study 1 do not constitute evidence for a mediating effect. Moreover, within study 1, only the three bivariate associations including social influence are based on experimental manipulation. The other three bivariate associations are cross-sectional. Accordingly, these latter associations give no information about causality, and therefore, the corresponding arrows in Figure 4 are not corroborated empirically. In study 2, as all six bivariate associations are cross-sectional, none of the arrows presented in Figure 6 are corroborated empirically. With the correlations presented in Table 6, any other order of the four investigated variables would produce a similar pattern as in Figure 6. In other words, for the given data, the authors’ statistical approach provides as much evidence for the investigated model as, for example, a model postulating that intention affects social influence and that this effect is mediated by performance expectation and credibility. The authors did not subject their model to any serious empirical test. Their study design precludes this. Causal relationships between cognitive and evaluative variables are very difficult to investigate. However, there are better study designs [2] than that applied by Guetz and Bidmon [1]. Moreover, one should give adequate consideration to methodological limitations when interpreting results.
